# MAIT cells are associated with responsiveness to neoadjuvant immunotherapy in COPD‐associated NSCLC

**DOI:** 10.1002/cam4.7112

**Published:** 2024-03-21

**Authors:** Yanze Yin, Ao Zeng, Abudumijiti Abuduwayiti, Zhilong Xu, Keyi Chen, Chao Wang, Xinyun Fang, Jiarui Wang, Gening Jiang, Jie Dai

**Affiliations:** ^1^ Department of Thoracic Surgery, Shanghai Pulmonary Hospital, School of Medicine Tongji University Shanghai China

**Keywords:** chronic obstructive pulmonary disease, immune checkpoint inhibitor therapy, lung cancer, mucosal‐associated invariant T cells, T cell exhaustion

## Abstract

**Background:**

Patients with non‐small cell lung cancer (NSCLC) and chronic obstructive pulmonary disease (COPD) experience worse clinical outcomes but respond better to immunotherapy than patients with NSCLC without COPD. Mucosal‐associated invariant T (MAIT) cells, a versatile population of innate immune T lymphocytes, have a crucial function in the response to infection and tumors. This study investigated the distribution of MAIT cells in COPD‐associated NSCLC and their involvement in the immune response.

**Methods:**

Flow cytometry, immunohistochemistry, and immunofluorescence were performed on tissue samples of patients with NSCLC, with or without COPD, treated with or without anti‐programmed death 1 (PD1) immunotherapy. MAIT cells were stimulated with 5‐OP‐RU using a mouse subcutaneous tumor model.

**Results:**

Tumors contained significantly more MAIT cells than paraneoplastic tissues, and CD8^+^ MAIT cells accounted for more than 90% of these cells. Patients with NSCLC and COPD had higher CD8^+^ MAIT cell counts than those with NSCLC without COPD. Additionally, patients with NSCLC and COPD displayed reduced expression of the activation marker, CD69, and functional markers, granzyme B (GZMB) and interferon γ (IFNγ), and higher expression of the immune exhaustion marker, PD1. Among patients who received immunotherapy, the proportion with a complete or partial response was higher in those with COPD than in those without COPD. In patients with NSCLC and COPD, the major pathologic response (MPR) group had higher MAIT levels than those in the no major pathologic response (NPR) group. In the mouse subcutaneous tumor model stimulation of MAIT cells using 5‐OP‐RU enhanced the antitumor effects of anti‐PD1.

**Conclusions:**

In patients with NSCLC and COPD, response to immunotherapy is associated with accumulation of CD8^+^ MAIT cells showing immune exhaustion. These findings may contribute to innovative approaches for immunotherapy targeting CD8^+^ MAIT cells.

## Graphical abstract

In patients with non‐small cell lung cancer (NSCLC), those with chronic obstructive pulmonary disease (COPD) are more responsive to immunotherapy. We compared mucosal‐associated invariant T cells (MAIT) levels and function in patients with NSCLC with and without COPD. Stimulation of MAIT cells using 5‐OP‐RU enhanced the effects of anti‐PD1 in mice. Response to immunotherapy was associated with the immune exhaustion of CD8^+^ MAIT cells.

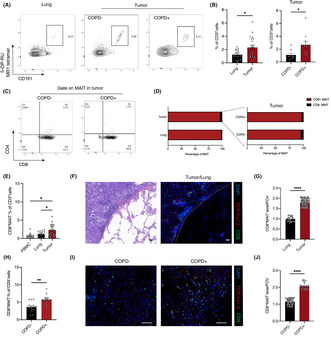

## 1 INTRODUCTION

Non‐small cell lung cancer (NSCLC) is a majority of lung cancer and is the leading cause of death due to cancer.^1^ Although the use of immunotherapy has led to marked improvements in patient survival, some patients are not sensitive or resistant to immune checkpoint inhibitor (ICI) therapy,^2^ the mechanism of which is unclear.

Chronic obstructive pulmonary disease (COPD) is a long‐term inflammation of lung that is primarily triggered by the inhalation of cigarette smoke or other air pollutants.^3^ The immune microenvironment of patients with pre‐existing COPD who subsequently develop NSCLC is unclear. Notably the presence of COPD is associated with enhanced effectiveness of ICI therapy in NSCLC.^4^ In spite of this, detailed investigations into the underlying mechanisms have not been carried out.

Mucosal‐associated invariant T cells (MAIT), which are specialized innate immune T cells, are distributed mainly in the mucous membranes and was initially found to have a significant role in infectious diseases.^5^, ^6^ Additionally, MAIT cells express rich immune checkpoints and have a powerful cytotoxic effect, thus playing an important role in antitumor immunology.^7^, ^8^ Cigarette smoking and COPD are associated with dysregulated activation of MAIT cells.^9^ Nonetheless, the involvement of MAIT cells in COPD‐associated NSCLC remains unclear.

The objective of this research was to investigate MAIT cells and their function in COPD‐associated NSCLC and simple NSCLC using flow cytometry. In addition, we compared the therapeutic efficacy of a control group, a MAIT activator group, an anti‐programmed death 1 (PD1) group, and an anti‐PD1+ MAIT activator group in animal studies. MAIT cells and their functions were analyzed in animal tumor samples using flow cytometry.

## 2 MATERIALS AND METHODS

### 2.1 Human tissues

All patients with NSCLC were collected from Shanghai Pulmonary Hospital between August 2020 and January 2023. All patients were provided with informed consent. Immunohistochemistry analysis of histopathology tissue slices after surgery confirmed the diagnosis of NSCLC. Individuals diagnosed with COPD were identified as those who had a forced expiratory volume in 1 s (FEV1)/forced vital capacity ratio less than 0.70 per second or exhibited clinically determined impaired pulmonary function. Clinical data on 342 NSCLC patients receiving neoadjuvant immunotherapy were collected. Tumor tissue from a cohort of 101 NSCLC patients (COPD−, *n* = 76; COPD+, *n* = 25) receiving neoadjuvant immunotherapy was collected for immunohistochemistry and immunofluorescence. Tumor tissue from another cohort of 26 NSCLC patients (COPD−, *n* = 15; COPD+, *n* = 11) receiving neoadjuvant immunotherapy was collected for flow cytometry. The research was carried out following the guidelines of the Declaration of Helsinki and received approval from the Institutional Ethics Committee at Shanghai pulmonary hospital (protocol code K23‐250). Table S1 contained a list of the clinical features of every patient.

### 2.2 Animal models

C57BL/6 mice were supplied by Shanghai Laboratory Animal Co., Ltd. in China. Experiments involve the utilization of male mice aged between 8 and 10 weeks. Approval for the animal study was acquired from the Institutional Animal Care and Use Committee of Shanghai Pulmonary Hospital.

For the mouse subcutaneous tumor model refer to the previous practice.^10^ 1 × 10^6^ Lewis cells in 120 μL PBS were injected subcutaneously into the C57BL/6 males in 8–10 weeks. 5‐OP‐RU is synthesized and used according to the previous description.^11^ Anti‐PD1 antibody and IgG control are used according to the previous description.^12^ Tumor tissues are collected in 20 weeks.

### 2.3 Flow cytometry

Flow cytometry experiments were conducted in accordance with the previously described protocol.^13^ Cells from human were incubated with the following: APC‐Cy7 live/dead (1:100, BD), PE‐Cy7 anti‐CD3 (1:100, BD), BV605 anti‐CD8 (1:100, BD), FITC anti‐T‐cell antigen receptor (TCR) Vα7.2 (1:200, BD), PerCP‐Cy5 anti‐ CD161 (1:100, BD), Human APC 5‐OP‐RU MR1 tetramer (ProImmune, 1:200), Pacific Blue anti‐PD1 (1:100, BD), AmCyan anti‐granzyme B (GZMB) (1:100, BD), PE anti‐interferon gamma (IFN‐γ) (1:100, BD). Mouse cells were incubated with BV510 anti‐CD45 (1:100, BD), BV421 5‐OP‐RU MR1 tetramer (National Institutes of Health tetramer facility, 1:300), APC anti‐TCR‐β (1:200, BD), PE anti‐PD1 (1:100, BD), PE‐Cy7 anti‐LAG3 (1:100, BD). The BD Fortessa FACS with Diva software v6.0 was utilized to conduct flow cytometry and data were processed using FlowJo V10 software.

### 2.4 Immunohistochemistry and immunofluorescence staining

The procedure previously^14^ was followed to conduct immunohistochemistry and immunofluorescence staining. Specifically, the primary antibody against CD8 (Abcam) was diluted at 1:100, the antibody against TCR Vα7.2 (Biolegend) was also diluted at 1:100, the antibody against GZMB (Abcam) was diluted at 1:100, the antibody against PD1 (Abcam) was also diluted at 1:100, the antibody against IL17A (Abcam) was diluted at 1:100, and the antibody against IFN‐γ (Abcam) was diluted at 1:100 for immunohistochemistry and immunofluorescence staining.

### 2.5 Statistical analysis

All statistical analyses were conducted with GraphPad Prism 7 software. The difference between two groups was compared using Mann–Whitney *U*‐test. *p* < 0.05 were considered statistically significant.

## 3 RESULTS

### 3.1 Abundance and distribution of MAIT cells in COPD‐associated NSCLC

**FIGURE 1:**
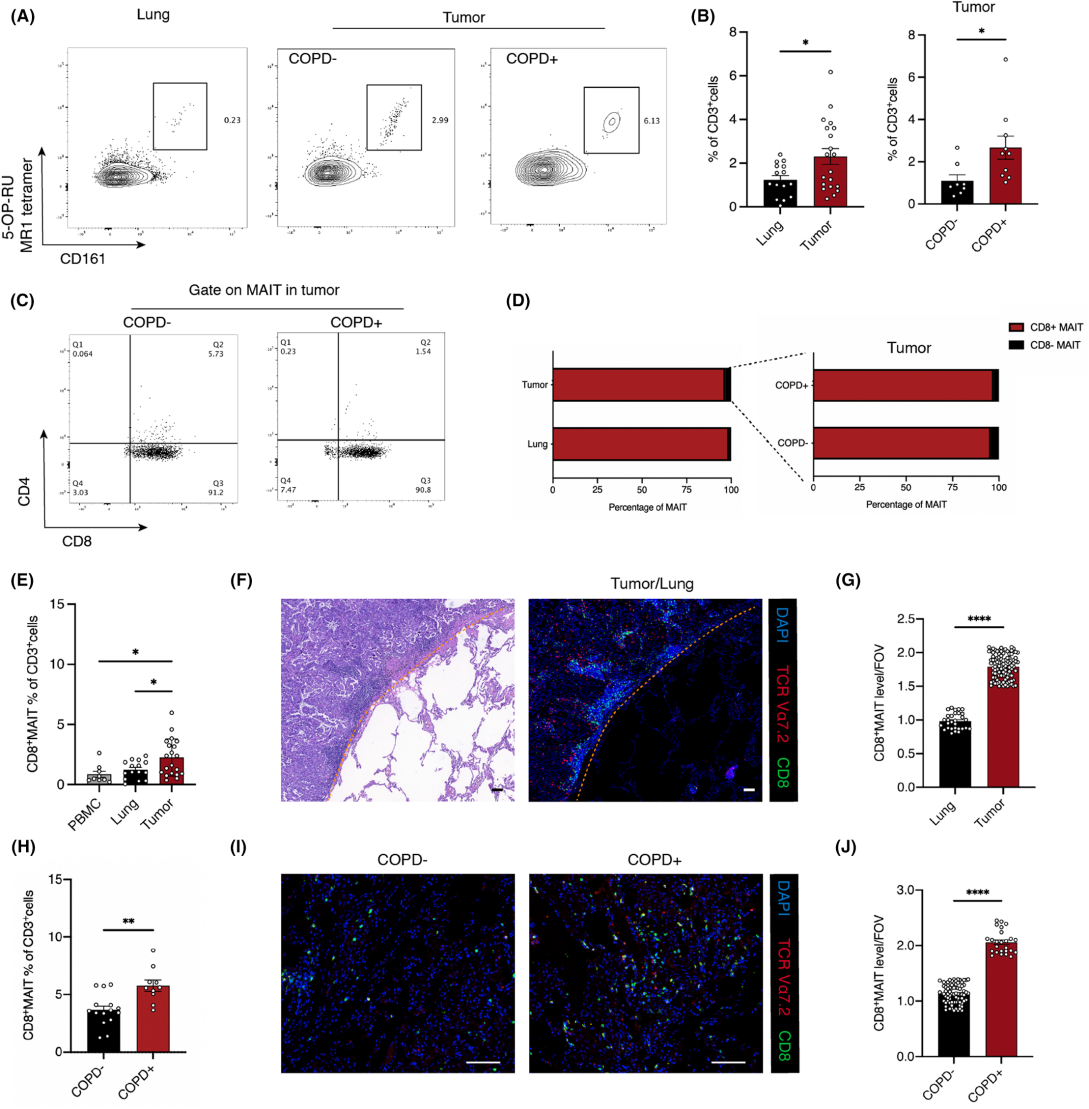
Abundance and distribution of MAIT cells in COPD‐associated NSCLC tumor tissues. (A, B) The frequency of MAIT cells in lung and tumor tissues, COPD− and COPD+ NSCLC tumor tissues. (A) Representative FCM figures and (B) statistical data. (C, D) Proportions of CD8^+^ MAIT cells in lung and NSCLC tumor tissues, COPD−, and COPD+ NSCLC tumor tissues. (C) Representative FCM figures and (D) statistical data. (E–G) Frequency of CD8^+^ MAIT cells in NSCLC. (E) Flow cytometry statistical data (PBMC, *n* = 9; lung, *n* = 15; tumor, *n* = 20), (F) representative immunofluorescence image. Scale bar, 100 μm. And (G) statistical data of immunofluorescence (lung, *n* = 30; tumor, *n* = 101). (H–J) The frequency of CD8^+^ MAIT cells in COPD− and COPD+ NSCLC tumor tissues. (H) Flow cytometry statistical data (COPD−, *n* = 15; COPD^+^, *n* = 10). (I) representative immunofluorescence image. Scale bar, 100 μm. (J) Statistical data of immunofluorescence (COPD−, *n* = 76; COPD^+^, *n* = 25). Lines and error bars are presented as the mean with SEM. *p < 0.05; **p < 0.01; ***p < 0.001 by Mann–Whitney *U*‐test. COPD, chronic obstructive pulmonary disease; MAIT, mucosal‐associated invariant T cells; NSCLC, non‐small cell lung cancer.

To investigate the distribution of MAIT cells in lung and NSCLC tumor tissue, we examined 20 NSCLC and paired lung tissue samples using flow cytometry and found that MAIT cells were significantly more abundant in tumor tissue than lung tissue. Additionally, we measured MAIT cells in 15 patients with NSCLC without COPD (COPD− NSCLC) and 12 patients with NSCLC and COPD (COPD+ NSCLC) and found that MAIT cells were more abundant in COPD+ compared to COPD− NSCLC tissue (Figure 1A,B). MAIT cells in peripheral blood are mostly CD8^+^ cells, therefore, we measured the proportion of CD8^+^ MAIT cells out of the total MAIT cells in NSCLC tumor and lung tissue. More than 90% of the MAIT cells were CD8^+^ cells in both lung and NSCLC tumor tissue, regardless of the patient's COPD status (Figure 1C,D). Furthermore, tumor had a higher percentage of CD8^+^ MAIT cells than lung tissue and PBMC, according to flow cytometry (Figure 1E). Immunofluorescence staining of tumor and lung tissue also showed more abundant CD8^+^ MAIT cells in tumor tissue than in lung tissue (Figure 1F,G). To explore the difference in the population of CD8^+^ MAIT cells between tissue samples from patients with COPD− and COPD+ NSCLC, we re‐analyzed the flow cytometry data. According to flow cytometry data, COPD+ NSCLC tissue had higher CD8^+^ MAIT cell populations than COPD− NSCLC tissue (Figure 1H). Immunofluorescence staining showed similar results (Figure 1I,J).

### 3.2 Function of CD8 ^+^ MAIT cells in COPD+ NSCLC

**FIGURE 2:**
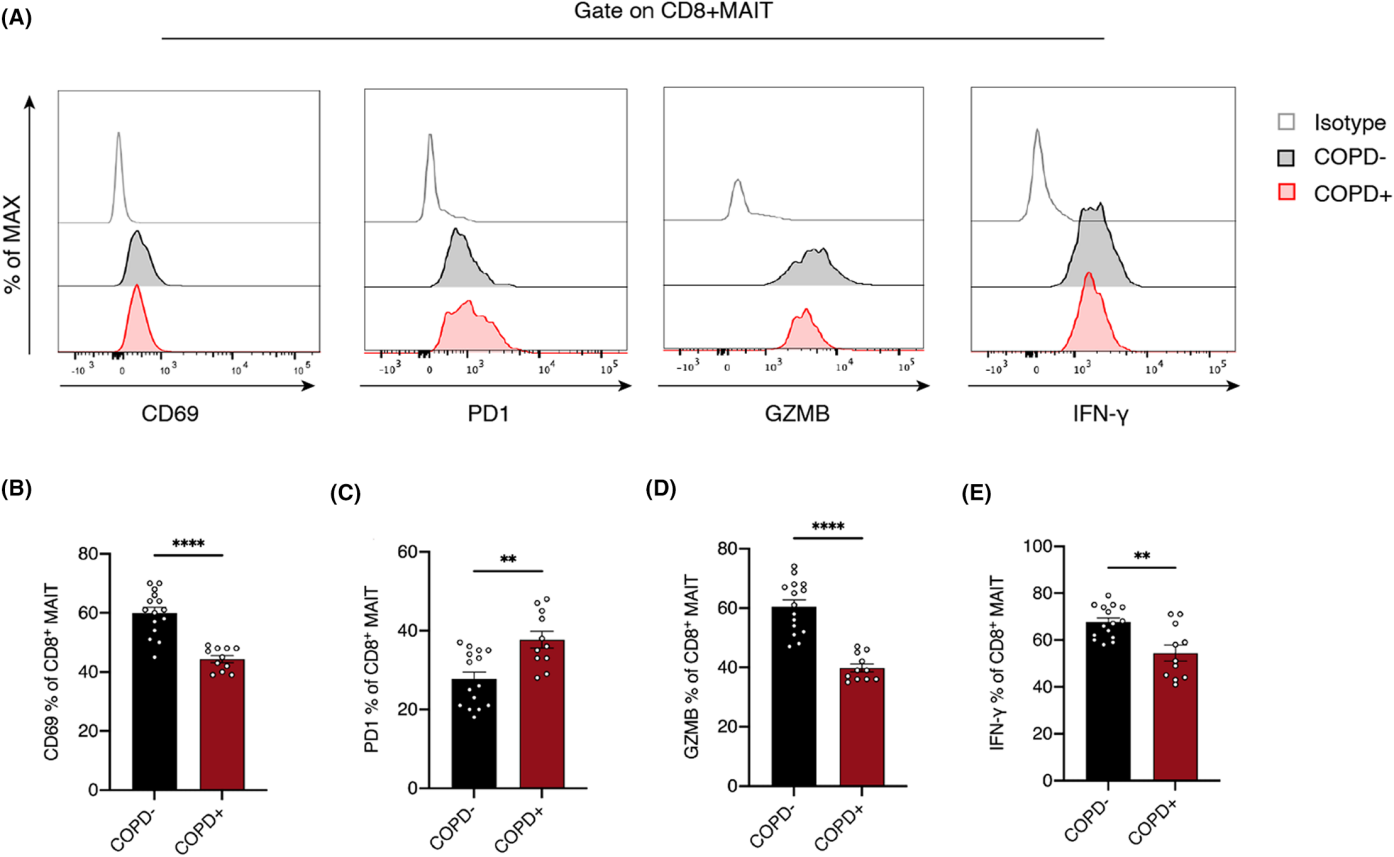
The functional phenotypes of CD8^+^ MAIT cells in COPD− and COPD^+^ NSCLC tumor tissues. (A) Activation marker CD69, immune exhausted marker PD1, cytotoxic markers GZMB and interferon gamma (IFN‐γ) are detected by flow cytometry. (B–E) Different expression level of CD69, PD1, GZMB, and IFN‐γ of CD8^+^ MAIT cells in COPD− and COPD^+^ NSCLC tumor tissues. Statistical data of (B) CD69, (C) PD1, (D) GZMB, and (E) IFN‐γ (COPD−, *n* = 15; COPD^+^, *n* = 10–11). Lines and error bars are presented as the mean with SEM. *p < 0.05; **p < 0.01; ***p < 0.001 by Mann–Whitney *U*‐test. COPD, chronic obstructive pulmonary disease; GZMB, granzyme B; MAIT, mucosal‐associated invariant T cells; NSCLC, non‐small cell lung cancer; PD1, programmed death 1.

To explore the status and function of CD8^+^ MAIT cells in COPD+ and COPD− NSCLC, we detected some important markers of CD8^+^ MAIT cells, including activation marker CD69, immune exhaustion marker PD1, cytotoxic markers GZMB and IFN‐γ, by flow cytometry (Figure 2A). Statistical analysis revealed that the expression of CD69, an activation indicator of CD8^+^ MAIT cells, was lower in COPD+ than COPD− NSCLC tissue (Figure 2B). The immune checkpoint PD1 of CD8^+^ MAIT cells was higher in COPD+ NSCLC tissue than in COPD− NSCLC tissue (Figure 2C), whereas the levels of GZMB and IFN‐γ in CD8^+^ MAIT cells was lower in COPD+ NSCLC tissue than in COPD− NSCLC tissue (Figure 2D).

### 3.3 MAIT cells are associated with responsiveness to immunotherapy in patients with NSCLC and COPD

**FIGURE 3:**
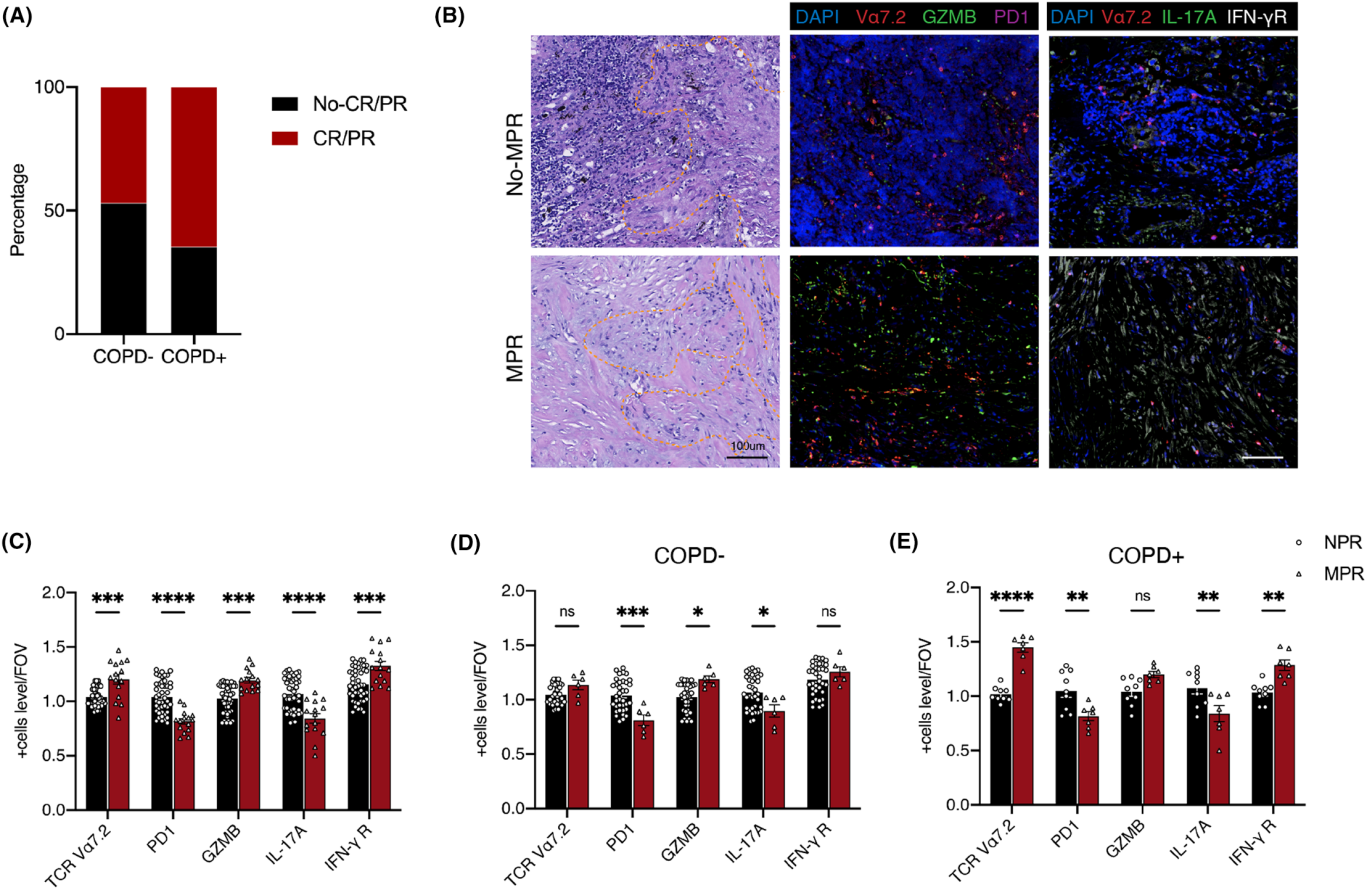
CD8^+^ MAIT cells are associated with the response of ICB therapy of non‐small cell lung cancer (NSCLC) patients. (A) CR/PR (COPD− NSCLC, *n* = 130; COPD^+^ NSCLC, *n* = 42) and no‐CR/PR (COPD− NSCLC, *n* = 147; COPD^+^ NSCLC, *n* = 23) rates of COPD− or COPD^+^ NSCLC patients receiving ICB therapy (COPD− NSCLC, *n* = 277; COPD^+^ NSCLC, *n* = 65; Total *n* = 342). (B) Representative immunofluorescence images of the MAIT cells distribution and PD1, GZMB, IL17A, and interferon γ receptor (IFNγ‐R) expression level in major pathologic response (MPR) and no‐major pathologic response (NPR) NSCLC patients. (C) The MAIT cells (TCR vα7.2) and expression level of PD1, GZMB, IL17A, and IFNγ‐R in total NSCLC patients receiving ICB therapy (NPR, *n* = 45; MPR, *n* = 15). (D) The MAIT cells (TCR vα7.2) and expression level of PD1, GZMB, IL17A, and IFNγ‐R in COPD− NSCLC patients receiving ICB therapy (NPR, *n* = 36; MPR, *n* = 6). (E) The MAIT cells (TCR vα7.2) and expression level of PD1, GZMB, IL17A and IFNγ‐R in COPD^+^ NSCLC patients receiving ICB therapy (NPR, *n* = 9; MPR, *n* = 7). Lines and error bars are presented as the mean with SEM. *p < 0.05; **p < 0.01; ***p < 0.001 by Mann–Whitney *U*‐test. COPD, chronic obstructive pulmonary disease; GZMB, granzyme B; MAIT, mucosal‐associated invariant T cells; PD1, programmed death 1; TCR, T‐cell antigen receptor.

We retrospectively analyzed data on 342 patients with NSCLC receiving preoperative neoadjuvant immunotherapy at Shanghai Pulmonary Hospital and found that patients with NSCLC and COPD had a better response to immunotherapy than those with NSCLC without COPD (Figure 3A). Next, we collected tissue samples from another cohort of patients with NSCLC receiving preoperative neoadjuvant immunotherapy and compared hematoxylin and eosin and immunofluorescence staining according to the patients' COPD status (Figure 3B). Compared with the no‐major pathologic response (NPR) group, the major pathologic response (MPR) group had a higher concentration of MAIT cells, lower PD1 levels, and higher GZMB levels. MAIT cells in the MPR group had lower interleukin 17A (IL‐17A) levels and higher interferon γ receptor (IFNγ‐R) levels (Figure 3C). In patients with NSCLC without COPD receiving preoperative neoadjuvant immunotherapy, the MPR subgroup showed no notable variation in the number of MAIT cells or levels of IFNγ‐R compared with the NPR subgroup, but had lower levels of PD1 IL‐17A and higher levels of GZMB (Figure 3D). In contrast, in patients with NSCLC and COPD receiving preoperative neoadjuvant immunotherapy, the MPR subgroup had a higher number of MAIT cells, higher levels of GZMB and IFNγ‐R, and lower levels of PD1 IL‐17A, than the NPR subgroup (Figure 3E).

### 3.4 CD8 ^+^ MAIT cells enhance the efficacy of anti‐PD1 therapy

**FIGURE 4:**
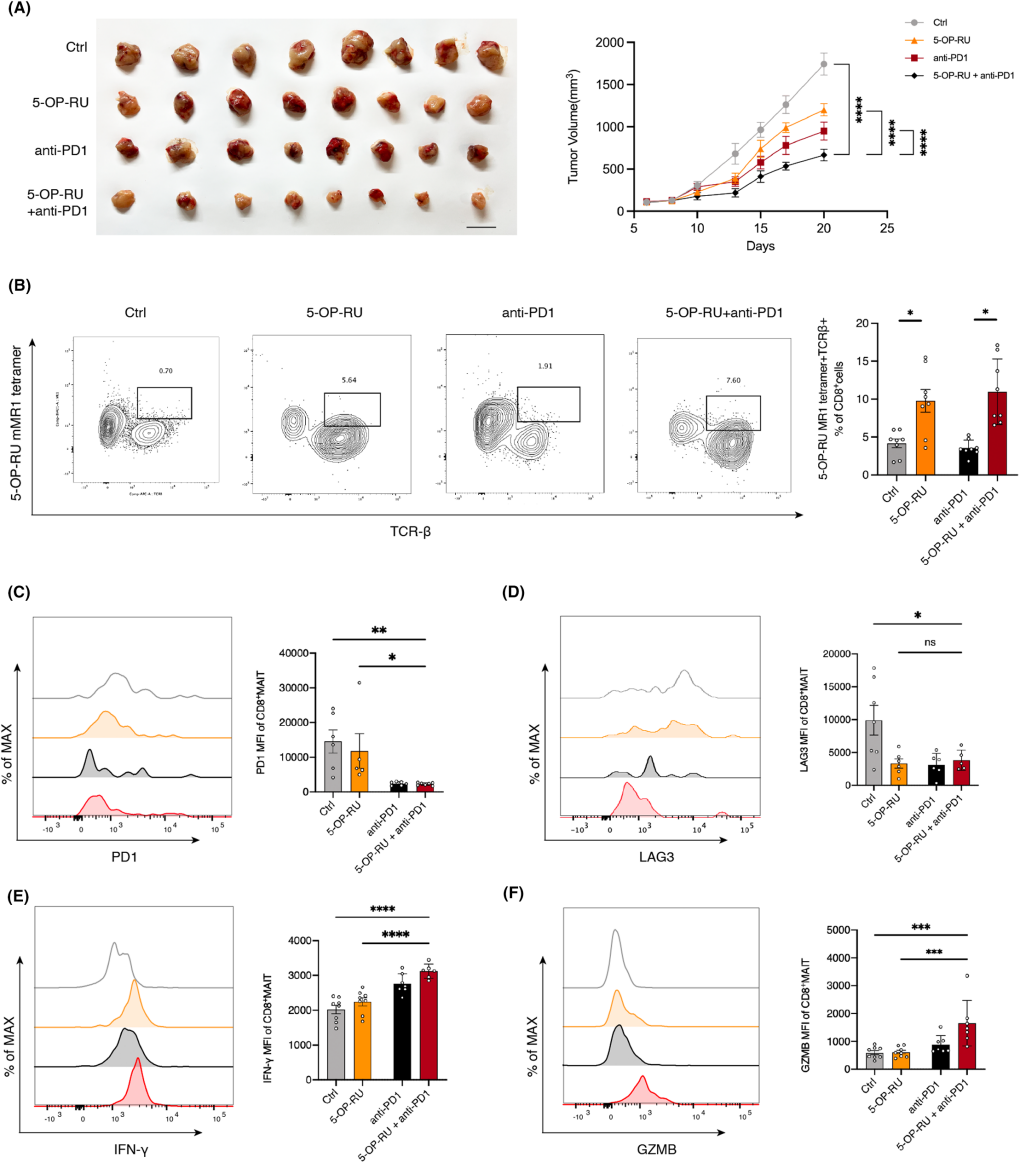
CD8^+^ MAIT cells enhance the efficacy of anti‐PD1. (A) 5‐OP‐RU enhances the tumor‐killing function of anti‐PD1 antibody in mice (*n* = 6–8 per group). (B) Proportion of CD8^+^ MAIT cells in TME is detected by flow cytometry with the treatment of 5‐OP‐RU, anti‐PD1 antibody and 5‐OP‐RU combined with anti‐PD1 antibody (*n* = 8 per group). (C–F) Functional markers of CD8^+^ MAIT cells detected by flow cytometry. Immune exhausted markers (C) PD1, (D) LAG3 and cytotoxic markers (E) interferon gamma (IFN‐γ), and (F) GZMB. (*n* = 6–8 per group). Lines and error bars are presented as the mean with SEM. *p < 0.05; **p < 0.01; ***p < 0.001 by Mann–Whitney *U*‐test. MAIT, mucosal‐associated invariant T cells; PD1, programmed death 1.

To investigate whether CD8^+^ MAIT cells can enhance the efficacy of immunotherapy, we established a tumor‐bearing mouse model receiving anti‐PD1 antibodies. 5‐OP‐RU was used to activate MAIT cells. The antitumor effects of anti‐PD1 antibodies were significantly enhanced by 5‐OP‐RU (Figure 4A). Flow cytometry analysis of tumor‐infiltrating immune cells demonstrated an increased percentage of CD8^+^ MAIT cells with 5‐OP‐RU treatment (Figure 4B). Consistently, we found an increased percentage of CD8^+^ MAIT cells in peripheral blood with 5‐OP‐RU treatment (Figure S1A). Additionally, we detected exhaustion of CD8^+^ MAIT cells in the microenvironment and found that 5‐OP‐RU combined anti‐PD1 antibodies induced lower PD1 expression level (Figure 4C). There was no significant difference in lymphocyte‐activation gene 3 (LAG‐3) levels between the 5‐OP‐RU combined anti‐PD1 antibody treatment group and the 5‐OP‐RU treatment group. However, both groups had markedly lower LAG‐3 levels than the control group (Figure 4D). The level of IFN‐γ in the group treated with both anti‐PD1 antibodies and 5‐OP‐RU was markedly higher than that in the control group and the group treated with only 5‐OP‐RU (Figure 4E). The GZMB level of the group treated with 5‐OP‐RU combined anti‐PD1 antibodies was also considerably higher than that of the control group and the 5‐OP‐RU group (Figure 4F). However, the PD1 and IFN‐γ levels of CD8^+^ MAIT cells in mouse blood treated with 5‐OP‐RU were not significantly different (Figure S1B,C). In Addition, we analyzed the phenotype of conventional CD8^+^ T cells in TME (Figure S2A–C).

## 4 DISCUSSION

MAIT cells have different effects on the prognoses of various cancers.^15^, ^16^, ^17^ This study confirmed that the proportion of MAIT cells was higher in NSCLC tumor compared to lung tissue and was dominated by CD8^+^ MAIT cells. A study on COPD found that the population of MAIT cells was higher in the lung tissue of patients with COPD compared to patients without COPD.^18^ Nevertheless, the role of MAIT cells in the tumor microenvironment of individuals with COPD remains unclear. In this study, the flow cytometry and immunofluorescence data demonstrated that the proportion of CD8^+^ MAIT cells in COPD+ NSCLC was higher than that in COPD− NSCLC. This phenomenon may be because MAIT cells monitor and respond to bacterial infections in patients with COPD.^19^ We also discovered that CD8^+^ MAIT cells in COPD+ NSCLC tissue had higher levels of the immune exhaustion marker PD1 and lower levels of the activation marker CD69 and functional markers GZMB and IFN‐γ than those in COPD− NSCLC tissue. These data suggest that CD8^+^ MAIT cells are highly enriched in NSCLC tissue of patients with COPD but have high levels of exhaustion and limited tumor‐killing effect. MAIT cells have been found to directly kill tumor cells and MAIT cells can produce tumor‐killing substances such as IFN‐γ and TNF.^7^, ^20^ Additional research is required to clarify the mechanisms that explain variations in the quantity and function of CD8^+^ MAIT cells in NSCLC tissue of patients with COPD.

Patients with COPD+ NSCLC have a worse overall prognosis than patients with COPD− NSCLC.^21^, ^22^ However, a recent study reported that patients with NSCLC and COPD have a higher objective response rate and longer progression‐free survival after receiving immunotherapy.^4^, ^23^ A review of a cohort of patients receiving preoperative neoadjuvant immunotherapy at our hospital revealed that the response rate among patients with NSCLC and COPD was considerably higher than that in patients with NSCLC without COPD. The immunofluorescence data showed that the concentration of MAIT cells was higher, the level of the immune exhaustion marker PD1 was lower, and the level of the tumor‐killing function marker GZMB was higher in the patients who responded to immunotherapy. Previous studies showed that the MAIT cell subfamily MAIT‐17 is mainly associated with immune non‐response, whereas MAIT‐IFNγ‐R is mainly associated with immune response.^24^ The results of this study also consistently showed that patients who responded to immunotherapy had lower IL‐17A levels and higher IFNγ‐R levels than those who did not respond to immunotherapy. These results suggest that MAIT cells are associated with responsiveness to immunotherapy in COPD+ NSCLC.

To confirm the causal relationship between MAIT cells and immunotherapy responsiveness in NSCLC, we activated CD8^+^ MAIT cells with 5‐OP‐RU using a mouse subcutaneous tumor model and found that the tumor‐killing effect of the anti‐PD1 antibody was significantly improved. Flow cytometry data showed that the immune exhaustion markers PD1 and LAG‐3 of CD8^+^ MAIT cells were significantly decreased in the 5‐OP‐RU combined anti‐PD1 antibody group, whereas the killing function markers IFNγ and GZMB were significantly increased. These results suggest that CD8^+^ MAIT cells can be activated by anti‐PD1 antibody to exert antitumor effects.

This study has some limitations, including a lack of long‐term follow‐up data. We cannot draw any conclusions about the long‐term effects of MAIT cells in patients receiving immunotherapy for NSCLC owing to a lack of data on survival due to the short follow‐up period. This cohort of patients require ongoing follow‐up to obtain long‐term prognostic data.

In conclusion, this study showed that MAIT cells were significantly enriched in the immune microenvironment of patients with NSCLC and COPD, and CD8^+^ MAIT cells were the main MAIT cells. However, CD8^+^ MAIT cells were in a state of immune exhaustion and thus had weak immune‐killing capacity. We demonstrated that anti‐PD1 antibody treatment can activate the immune‐killing function of CD8^+^ MAIT cells in patients with NSCLC and COPD, and responsiveness to immunotherapy is related to the distribution of MAIT‐17 and MAIT‐IFNγ‐R cell subclusters. These results reveal possible mechanisms underlying the better response to immunotherapy in patients with NSCLC and COPD, demonstrate the potential of MAIT cells as a predictive indicator for immunotherapy in patients with NSCLC, and may contribute to new strategies to improve the effectiveness of immunotherapy in NSCLC.

## AUTHOR CONTRIBUTIONS

**Yanze Yin:** Data curation (equal); formal analysis (equal); investigation (equal); project administration (equal); writing – original draft (lead). **Ao Zeng:** Data curation (equal); formal analysis (equal); project administration (equal); writing – original draft (equal). **Abudumijiti Abuduwayiti:** Data curation (equal); project administration (equal). **Zhilong Xu:** Data curation (equal); project administration (equal). **Keyi Chen:** Data curation (equal); project administration (equal). **Chao Wang:** Data curation (equal); project administration (equal). **Xinyun Fang:** Data curation (equal); project administration (equal). **Jiarui Wang:** Data curation (equal); project administration (equal). **Gening Jiang:** Conceptualization (equal); writing – review and editing (equal). **Jie Dai:** Conceptualization (equal); writing – review and editing (equal).

## FUNDING INFORMATION

This study was supported by the National Natural Science Foundation of China (82172848) and Shanghai Pulmonary Hospital Fund (fkzr2307, fkzr2105, and fkyq1908).

## CONFLICT OF INTEREST STATEMENT

The authors have no conflict of interest.

## ETHICS STATEMENT

Approval of the research protocol by an Institutional Reviewer Board: the Institutional Ethics Committee at Shanghai pulmonary hospital. Registry and the registration no. of the study/trial: (protocol code K23‐250). Animal studies: Approval for the animal study was acquired from the Institutional Animal Care and Use Committee of Shanghai Pulmonary Hospital.

## Supporting information


Figure S1.

Figure S2.

Table S1.

## Data Availability

These data generated during the study are available from the corresponding author on reasonable request.
