# Supplementary figures and images for: MAIT cells are associated with responsiveness to neoadjuvant immunotherapy in COPD‐associated NSCLC
Source: Cancer Med. 2024 Mar 21;13(6):e7112. doi: 10.1002/cam4.7112 (PMC10955227; doi:10.1002/cam4.7112)

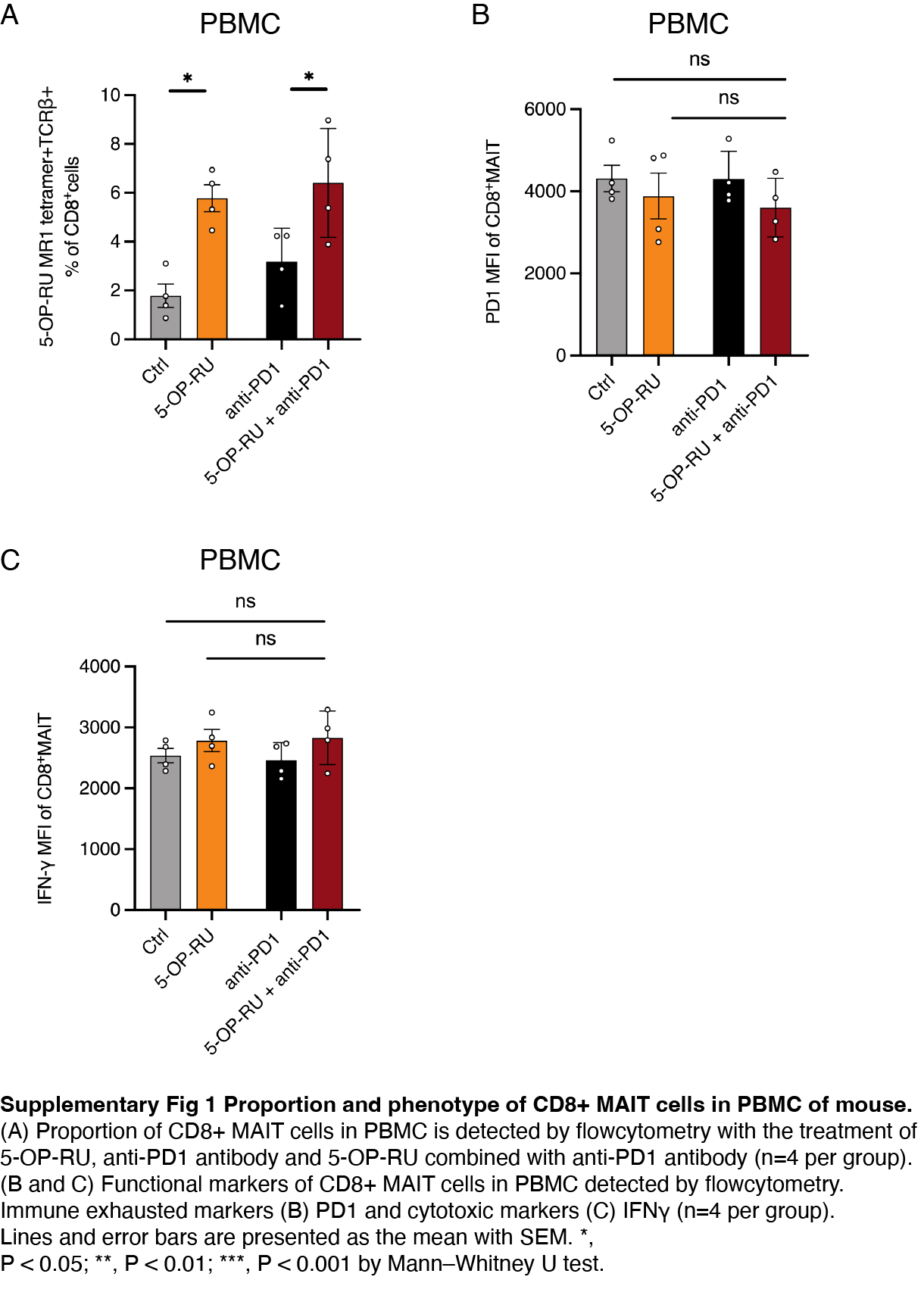

Supplement: Supplementary file 1 — Figure S1. [file CAM4-13-e7112-s001.tif]

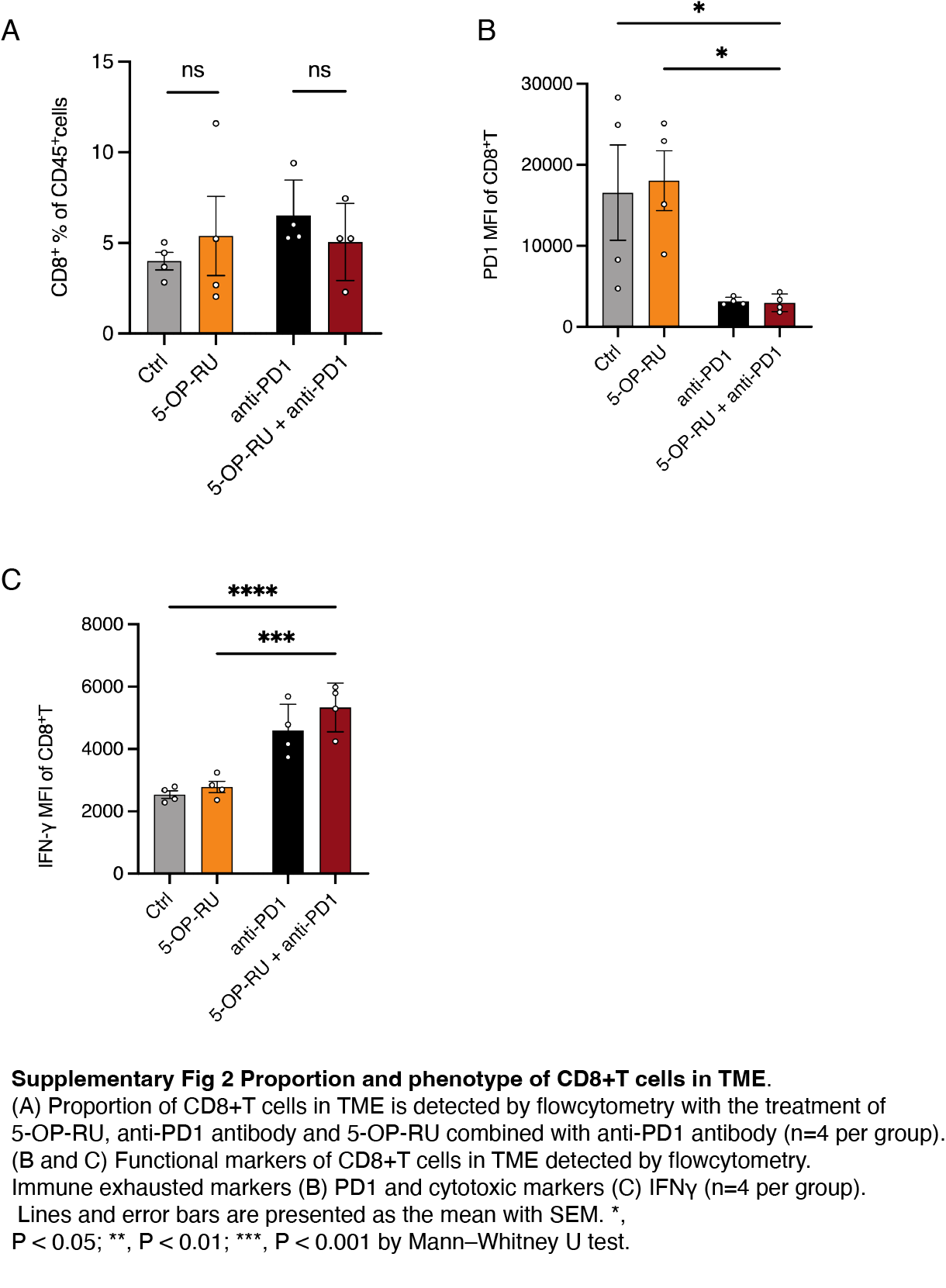

Supplement: Supplementary file 2 — Figure S2. [file CAM4-13-e7112-s003.tif]
