# Supplementary material for: MAIT cells are associated with responsiveness to neoadjuvant immunotherapy in COPD‐associated NSCLC
Source: Cancer Med. 2024 Mar 21;13(6):e7112. doi: 10.1002/cam4.7112 (PMC10955227; doi:10.1002/cam4.7112)
Supplement: Supplementary file 3 — Table S1. [file CAM4-13-e7112-s002.docx]

| **Table 1.**  **Clinical features of patients with NSCLC receiving neoadjuvant immunotherapy** | | | |
| --- | --- | --- | --- |
|  | **Total** | **COPD- NSCLC** | **COPD+ NSCLC** |
|  | **n=342** | **n=277** | **n=65** |
| **Gender** |  |  |  |
| **Male** | 314 (91.8) | 251 (90.6) | 63 (96.9) |
| **Female** | 28 (8.2) | 26 (9.4) | 2 (3.1) |
| **Age** |  |  |  |
| **≤60** | 134 (39.2) | 117 (42.2) | 17 (26.2) |
| **＞60** | 208 (60.8) | 160 (67.8) | 48 (73.8) |
| **Pathology** |  |  |  |
| **LUAD** | 87 (25.4) | 68 (24.5) | 19 (29.2) |
| **LUSC** | 212 (62.0) | 171 (61.7) | 41 (63.1) |
| **Others** | 43 (12.6) | 38 (13.8) | 5 (7.7) |
| **Clinical stage** |  |  |  |
| **I/II** | 58 (17.0) | 42 (15.2) | 16 (24.6) |
| **III/IVA** | 284 (83.0) | 235 (84.8) | 49 (75.4) |
| **Smoking history** |  |  |  |
| **No** | 19 (5.5) | 18 (6.5) | 1 (1.5) |
| **Yes** | 262 (76.8) | 209 (75.5) | 53 (81.5) |
| **Unknown** | 61 (17.7) | 50 (18.0) | 11 (17.0) |
| **PD-L1 expression** |  |  |  |
| **PD-L1<50%** | 188 (55.0) | 154 (55.6) | 34 (52.3) |
| **PD-L1≥50%** | 49 (14.6) | 40 (14.4) | 9 (13.8) |
| **Unknown** | 105 (30.4) | 83 (30.0) | 22 (33.9) |
| Values are presented as n (%); NSCLC, non-small cell lung cancer; LUSC, lung squamous cell carcinoma; LUAD, lung adenocarcinoma; COPD, chronic obstructive pulmonary disease; PD-L1, programmed cell death ligand 1 | | | |
